# The Canadian retinoblastoma research advisory board: a framework for patient engagement

**DOI:** 10.1186/s40900-020-0177-8

**Published:** 2020-02-28

**Authors:** Maxwell J. Gelkopf, Iva Avramov, Richelle Baddeliyanage, Ivana Ristevski, Sarah A. Johnson, Kaitlyn Flegg, Helen Dimaras

**Affiliations:** 1 Department of Ophthalmology & Vision Sciences, The Hospital for Sick Children, 555 University Ave, Toronto, ON M5G 1X8 Canada; 2 Child Health Evaluative Sciences Program, SickKids Research Institute, Toronto, Canada; 3 Human Biology Program, Faculty of Arts & Science, University of Toronto, Toronto, Canada; 4 Department of Neuroscience, Evelyn F. and William L. McKnight Brain Institute, University of Florida, Gainesville, Florida USA; 5 Division of Clinical Public Health, Dalla Lana School of Public Health, University of Toronto, Toronto, Canada; 6 Department of Ophthalmology & Vision Sciences, Faculty of Medicine, University of Toronto, Toronto, Canada; 7 Department of Human Pathology, College of Health Sciences, University of Nairobi, Nairobi, Kenya

**Keywords:** Retinoblastoma, Patient engagement, Patient and Public involvement (PPI), Research engagement, Advisory board, Patient-oriented research

## Abstract

**Plain English summary:**

Retinoblastoma is a rare eye cancer that occurs in one or both eyes of infants and young children as a result of errors in the *RB1* gene. There are approximately 2000 retinoblastoma survivors in Canada. Those with the heritable form of the disease are at risk of passing the gene to the next generation and developing a second cancer. Many retinoblastoma survivors and families therefore interact with the healthcare system throughout their lives.

The retinoblastoma community has a longstanding history of engaging patients in research, however without any formal process. The literature establishes benefits to patient engagement such as research results which are more applicable, credible, and transparent. Building on the established interest among stakeholders, the Canadian Retinoblastoma Research Advisory Board (CRRAB) was established in 2016 to foster sustainable and meaningful collaboration between patients (survivors and family members), advocacy groups, healthcare professionals, and researchers in the retinoblastoma community.

The aim of this study was to evaluate the utility of CRRAB in fostering patient engagement in research. Members of CRRAB were surveyed to uncover their attitudes towards and experience with patient engagement in research. Participants perceived CRRAB to provide diverse and accessible opportunities for patient engagement in research and perceived their participation to have a meaningful impact. The results suggest that CRRAB promotes patient engagement in retinoblastoma research, and provides direction to sustain and enhance future patient engagement.

**Abstract:**

**Background**

The Canadian Retinoblastoma Research Advisory Board (CRRAB) is a multidisciplinary group, including patients (survivors and family members), advocacy groups, healthcare professionals, and researchers, which aims to establish and sustain patient engagement in retinoblastoma research. The purpose of this study was to describe the development of CRRAB and to uncover members’ understanding of and attitudes towards patient engagement in research. As well, to determine their level of engagement.

**Methods**

Retinoblastoma patients, healthcare professionals, and researchers provided leadership to co-develop CRRAB. CRRAB members were surveyed by pre- and post-test questionnaire at the 2016 Annual General Meeting to assess experience with, understanding of, and attitudes towards patient engagement in research. A second questionnaire was administered before the 2017 CRRAB meeting to assess awareness and perceived impact of CRRAB activities, and individual engagement in research. Data were analyzed by descriptive statistics and paired t-test (for pre/post-test). Thematic analysis of chart board discussions at both meetings revealed the joint goals of CRRAB and reasons for and barriers to patient engagement.

**Results**

In 2016, 21 individuals participated and self-identified as patients (11, 52%), healthcare professionals (6, 29%), and/or researchers (7, 33%) (participants could overlap stakeholder groups). Overall, participants believed that research is relevant to all stakeholders and that patients can have meaningful impact on research. In 2017, 35 individuals participated and identified as patients (21, 60%), healthcare professionals (9, 26%), and/or researchers (8, 23%). 94% of participants were aware of CRRAB initiatives and 67% had participated in at least one over the previous year. Participants perceived that CRRAB provides diverse opportunities and increases accessibility for patient engagement in research, and perceived patient engagement to have meaningful impact on retinoblastoma research. Chart board discussions revealed that participants wanted to be part of CRRAB to increase knowledge, support innovation and patient engagement, and be part of a community. Members most commonly faced barriers including time and cost restraints.

**Conclusions**

The results of this study suggest that CRRAB has supported the engagement needs of patients affected by retinoblastoma, and has provided an opportunity for engaging patients in retinoblastoma research. CRRAB will continue to be used as a framework for patient engagement, with improvements based on participant feedback.

## Introduction

Retinoblastoma (RB) is a childhood cancer of the eye that is newly diagnosed in 25 children each year in Canada [1]. A biallelic mutation in the *RB1* gene leads to the development of a tumour in one or both eyes [1]. Children may suffer visual impairment in one or both eyes as a consequence of disease progression and treatment [1]. Upon diagnosis parents and caregivers are faced with understanding the complicated disease and its genetic implications [2]. Given that retinoblastoma can be caused by somatic or germline mutations, patients and their families must undergo genetic testing to determine their risks of passing the disease trait onto their offspring. As well, patients with germline mutations have risk of developing second cancers later in life. For these reasons, retinoblastoma not only affects a child at the time of diagnosis, but also throughout their entire life with extended effects related to prognosis, treatment, family planning, and second cancers. There are an estimated 2000 retinoblastoma survivors in Canada, each requiring extended clinical follow-up and long-term interaction with the healthcare community; however, it is not clear if these individuals remain aware of their childhood diagnosis or connected to a follow-up clinic.

Patient and Public Involvement (PPI) in research is that which is carried out ‘with’ or ‘by’ patients and the public, as opposed to research conducted ‘on’ or ‘about’ these individuals [3]. In Canada, PPI goes by the name of ‘Patient Engagement’, a concept that similarly shifts the idea of a patient from a passive data-point to an active participant who is encouraged to be involved in any or all aspects of the research process [4]. The Canadian Strategy for Patient-Oriented Research (SPOR) promotes patient engagement as a meaningful and active partnership between patients and healthcare professionals in governance, priority setting, conducting research, and knowledge translation [5]. In line with this notion, the SPOR definition of the term ‘patient’ to includes individuals with lived experience of disease, including family members and informal caregivers [6].

The literature establishes that the benefits of patient engagement in research are that study findings are often more applicable, credible and transparent [7]. Moreover, there are benefits to the patients themselves, such as heightened autonomy, dignity, and self-worth [7, 8]. Finally, patient engagement in research mediates difficulty in effectively translating research results into clinical practice and public health policy [4].

Given the genetic and other long-term implications of a retinoblastoma diagnosis, patients are incentivized to contribute to, co-create, and keep abreast of retinoblastoma research. The retinoblastoma community in Canada has historically been a strong advocate for patient engagement in clinical care, as evident by patient involvement in developing the first clinical retinoblastoma guidelines [9]. The Canadian Retinoblastoma Patient Engagement Strategy was established to address the needs of patients and create a sustainable avenue for meaningful and accessible patient engagement in research [10]. The specific aims of this Strategy were to: i) share research results with those affected by retinoblastoma; ii) include a diverse group of individuals affected by retinoblastoma in research; and iii) promote research created and led by those affected by retinoblastoma. The Canadian Retinoblastoma Research Advisory Board (CRRAB) was created alongside patients to govern and sustain the national patient engagement strategy [10]. Here we report on the development of CRRAB and the evaluation of its first 2 years of operation.

## Methods

### Aims

The primary aim of this study was to describe the development of CRRAB and to evaluate the effectiveness of CRRAB as a framework for patient engagement in research. The secondary aim was to uncover members’ understanding of and attitudes towards patient engagement in research, and level of research engagement in ongoing CRRAB activities.

### Design

This was a longitudinal, non-randomized study. This study used mixed qualitative and quantitative methods and adheres to the Guidance for Reporting Involvement of Patients and Public-2 (GRIPP2) [11] (Additional file 1).

### Development of CRRAB

A national multidisciplinary group, CRRAB was established, following SPOR’s guidelines [3], to govern and sustain the strategy, fostering an environment for retinoblastoma patients, healthcare professionals, and researchers to develop meaningful, co-directed research that is relevant to patients and improves health outcomes [10].

#### Patient and Public involvement

Guidance and support were provided by retinoblastoma patients, healthcare professionals, and researchers throughout the development of the national patient engagement strategy. Specifically, focus group studies were held in Toronto and Calgary to determine how to best engage patients throughout the research process and to address potential barriers (publication pending). Representatives of the patient advocacy groups, World Eye Cancer Hope (www.wechope.org) and The Canadian Retinoblastoma Society (https://www.rbsociety.ca/), along with 3 additional patient partners, served as collaborators on a grant to obtain seed funding for the initial activities of the strategy, including the first CRRAB meeting. These patient partners were involved in the design, development and initial implementation of CRRAB and continue to guide and direct CRRAB in leadership roles and assist with research dissemination. Patients, healthcare professionals and researchers select their own time commitment and level of involvement (i.e., attend meetings, provide feedback on progress, make decisions, take on a leadership position, etc.).

#### CRRAB member recruitment

Retinoblastoma patients, including family members and caregivers, healthcare professionals (including clinicians, allied health providers, and their trainees), researchers, policy makers, and patient engagement experts in Canada were eligible to become members of CRRAB. Retinoblastoma healthcare professionals around Canada were invited to participate, and were also asked to invite their current and past patients to join CRRAB. The Canadian Retinoblastoma Research Registry, a nationwide patient registry, was established as part of the national patient engagement strategy and was used to recruit patients to CRRAB [12]. At the time of the second CRRAB meeting, the registry had 40 members with distribution across Canada [12]. Participants were also recruited by social media, written correspondence, word of mouth, and email. Recruitment began on October 10th, 2016, with an aim of including a diverse group of participants, comprised of 50% or greater patients.

### Evaluation of CRRAB: data collection and analysis procedures

To evaluate the effectiveness of CRRAB as a framework for patient engagement, members were asked to assess their experience with, understanding of, and attitudes towards patient engagement in research. Members were asked to complete questionnaires at multiple time points during the two annual general meetings and to participate in group discussions at these meetings to reflect on their involvement with the advisory board. As this is an ongoing patient engagement strategy, study methodology has evolved between annual meetings. Early evaluation helped identify attitudes and preferences that shaped CRRAB. These changes allowed a better understanding of patient engagement through the lens of CRRAB.

#### First annual CRRAB meeting

The first annual CRRAB meeting took place on December 3rd, 2016 at the Hospital for Sick Children in Toronto, Canada.

##### Pre- and post-meeting questionnaire

A questionnaire was distributed to CRRAB meeting attendees to be completed before and after the meeting (Additional file 2). Questions asked for (i) demographic information (pre-meeting questionnaire only), and (ii) experience with, understanding of, and attitudes towards patient engagement (pre- and post-meeting questionnaire). The questionnaire was modeled after existing studies that explored patient engagement in research [13, 14]. The pre- and post-meeting results were analyzed using a paired t-test in IBM® SPSS® Statistics Version 25 [15].

For questions 1–3, expected results were towards a value of 5 (strongly disagree). For questions 4–6, expected results were towards a value of 1 (strongly agree). Those that did not complete both the pre- and post-test questionnaires had both their questionnaires excluded from data analysis.

For quantitative results statistical significance was set at *p* < 0.05. For all Likert data, mean, median, mode, and range were calculated.

##### Chart board discussions

During the meeting, participants were asked to contribute to group discussions and provide answers to questions on a chart board (Additional file 2). These questions were designed to gather information further information on experience with, understanding of, and attitudes towards patient engagement. The first question asked participants to share their goals of participating in CRRAB; the second solicited specific research questions about retinoblastoma; and the third question asked participants to suggest methods to better engage patients. These results were analyzed using qualitative methods to extract common themes in NVivo® Version 12 [16]. Statements could be classified under more than one theme. When the research team was unable to make a conclusive decision on a response’s meaning due to fragmented sentences, it was excluded. Coding was iterative (repeated systematically) and reviewed together by authors to ensure unbiased results.

#### Second annual CRRAB meeting

The second annual CRRAB meeting took place on December 10th, 2017 in the same location. The second annual general meeting was held in conjunction with a Retinoblastoma Family Gathering and a priority setting workshop. During the family gathering CRRAB members, their families, and other stakeholders were invited to attend presentations given by healthcare professionals and patients, and a marketplace showcasing organizations offering relevant resources. The priority setting workshop was a full-day activity to determine the “top 10” retinoblastoma research priorities in Canada (publication pending).

##### Pre-meeting questionnaire

A pre-meeting questionnaire was distributed to all CRRAB members before the annual meeting (Additional file 3). As such, not all participants that completed the questionnaire were present at the second annual CRRAB meeting. The questionnaire asked participants to provide (i) demographic information, (ii) information on awareness and perceived impact of CRRAB activities, and individual engagement in research, and (iii) opinions on the goals of CRRAB. This questionnaire was developed based on expert opinions of the study team.

##### Chart board discussions

Participants were asked to contribute to group discussions and provide answers to chart board questions (Additional file 3). These questions evolved from the first meeting and were designed to determine members’ understanding of the purpose of CRRAB and plan future directions. The first question asked participants to propose ideas for future CRRAB goals and activities. The second question asked for participants’ ideas of how to increase patient and family leadership. The third and fourth questions asked participants why they do and do not want to be part of CRRAB working groups. The last question asked participants to share their perceptions of the purpose of CRRAB These results were analyzed using qualitative methods to extract common themes in NVivo® Version 12 [16]. Multiple responses could be classified under more than one theme. Similar to the first annual CRRAB meeting, coding was iterative, conducted in a group setting, and responses that were inconclusive were excluded.

##### Post-meeting questionnaire

At the conclusion of the CRRAB meeting, a questionnaire was distributed to evaluate participant satisfaction with the meeting on a five-point Likert scale (Additional file 3). In addition to overall satisfaction, participants were asked how likely they were to recommend CRRAB, if their objectives were met, what they liked most, what could be improved, and if the next steps for CRRAB were clear.

For all quantitative results statistical significance was set at *p* < 0.05. For the Likert data, mean, median, mode, and range were calculated.

## Results

### The Canadian retinoblastoma research advisory board

#### CRRAB membership

CRRAB is a multidisciplinary group consisting of retinoblastoma patients (survivors and family members), healthcare professionals (physicians and allied health providers), researchers, and other relevant stakeholders.

#### CRRAB structure

CRRAB is governed by a steering committee, three working groups (WG) and general members. Members of CRRAB meet annually, while the three working groups, Patient Engagement, Research Advisory, and Research Development, meet via videoconference every 4–6 weeks. WGs are composed of 5–10 individuals, and led by patient and non-patient co-chairs.

The Patient Engagement WG is responsible for identifying and including a diverse group of patients in the Canadian Retinoblastoma Research Registry. The Research Advisory WG governs the research registry and connects patients with research opportunities and findings, for example through publication of a quarterly blog. The final group, the Research Development WG, is designing a research proposal based on one of the “top 10” retinoblastoma research priorities (publication pending). The Research Development WG was formerly the Priority Setting WG, which established the “top 10” retinoblastoma research priorities shared amongst patients, healthcare professionals and researchers.

Although certain aims and activities are divided between the WGs, there is continuous communication between groups, and membership is not limited to one WG.

CRRAB members self-identified as patients, healthcare professionals, researchers and/or other. All individuals were eligible to be elected to leadership roles to further patient engagement and create a self-sustainable advisory board.

Retinoblastoma patients were also given the opportunity to become an RB Champion, with training and resources to help to share their story and promote CRRAB initiatives.

#### CRRAB social media and online engagement

CRRAB recruitment began with establishing social media platforms and online engagement tools. CRRAB initially began with a patient engagement website (www.rbresearch.ca), Twitter profile (www.twitter.com/rb_research) and a Facebook page (www.facebook.com/RBresearch/). These platforms were used to recruit CRRAB members and inform individuals about the Retinoblastoma Research Registry. The Research Advisory Working Group then created the Canadian Retinoblastoma Research Website (www.rbcanadaresearch.com). The website houses information about CRRAB, the RB champion program, and a quarterly blog. The blog posts – written by patients, healthcare professionals, and researchers – showcase research opportunities and results, CRRAB accomplishments, and information about retinoblastoma. The blog content is also emailed to registrants.

#### Alignment of CRRAB with SPOR

The SPOR Framework, published in 2014, identified four guiding principles to meaningfully engage patients in research: inclusion, support, mutual respect and co-building [5]. To promote the inclusion of a diverse and representative group of patients, CRRAB offered travel bursaries and made accommodations for participants as required. To support CRRAB members in contributing freely to discussions, CRRAB promoted a welcoming environment with an emphasis on education, collaboration and protection of privacy. To ensure mutual respect, members used first names and excluded formal titles to promote equal partnership, and were encouraged to contribute to discussions at any point. To practice co-building – members working together to identify and execute goals [5] – individuals were elected to a governance structure, and together determined the future goals of CRRAB. Evidently, the four guiding principles of the SPOR were critical elements of the CRRAB’s framework. Taken together, CRRAB emerged as a model for applying meaningful patient engagement in scientific research and the broader healthcare system.

### Participant demographics

Participants from Ontario, Alberta, Québec, and Nova Scotia joined the first annual CRRAB meeting in person and via teleconference (n=22, Table 1). At the first annual CRRAB meeting three participants identified as survivors and eight identified as parents of children diagnosed with retinoblastoma. Together, 50% (11/22) of participants were individuals with a personal experience of retinoblastoma (patients as defined by SPOR) [5]. Out of the three survivors, one was diagnosed 30–39 years ago, and two were diagnosed 40–49 years ago. One survivor was also a parent to a child with retinoblastoma. Those that were parents (*n* = 8) had either one child (*n* = 7) or three children (*n* = 1) diagnosed with retinoblastoma. Their children (*n* = 10) were diagnosed with a range of less than 1 year ago (1/10 10.00%) to 10+ years ago (1/10, 10.00%), with the majority diagnosed 1–5 years ago (8/10, 80.00%). Six participants (6/22, 28.57%) were healthcare professionals (doctors, nurses, social workers etc.) and six (6/22, 28.57%) were researchers. Two (2/22, 9.09%) classified themselves as ‘other’, with involvement related to patient engagement activities. CRRAB members learned about the first annual CRRAB meeting most commonly from a member of the research team (10/22, 45.45%), followed second most often by email (7/22, 31.82%).

**Table 1:** Demographics of CRRAB members at the time of the first and second annual CRRAB meetings: total participants, participant types, previous involvement and how they first learned about CRRAB

	First Annual Meeting		Second Annual Meeting	
	n	%	n	%
Total Participants	22	100.00	35	100.00
Participant Type				
Patient (Survivor or Parent)	11	50.00	21	60.00
Healthcare Professional	6	27.27	9	25.71
Researcher	6	27.27	8	22.86
Other	2	9.09	0	0.00
*Note: individuals can be part of more than one group				
Involvement (2017 CRRAB Meeting Only)				
Attended CRRAB 2016			10	28.57
Registered for CRRAB 2017			27	77.14
Not involved previously			14	40.00
Involved less than 1 month			2	5.71
Involved 1–3 months			2	5.71
Involved 3–6 months			4	11.43
Involved 6–9 months			3	8.57
Involved 9–12 months			10	28.57
Timing of RB Diagnosis (2016 CRRAB Meeting Only)				
Survivor (*n* = 3)	3	100.00		
*30–39 years ago*	1	33.33		
*40–49 years ago*	2	66.67		
Parents of Children (*n* = 8)	8	100.00		
Children of Participants (*n* = 10)	10	100.00		
*less than 1 year ago*	1	10.00		
*1–5 years ago*	8	80.00		
*10+ years ago*	1	10.00		
How they heard about CRRAB				
Research Team	10	45.45	22	62.86
Healthcare Professional	1	4.55	6	17.14
Email/Pamphlet/Social Media	7	31.82	5	14.29
Co-Worker	3	13.64	0	0.00
Family Member	1	4.55	1	2.86
Patient	0	0.00	1	2.86
Family Advisory Network at SickKids	1	4.55	0	0.00

*statement was classified under more than one theme

Individuals from Ontario, Alberta, British Columbia, Québec, Nova Scotia, and Manitoba participated in the second annual CRRAB meeting in-person. Thirty-five CRRAB members completed the questionnaire before the second annual CRRAB meeting: 21 were patients (60%), 9 were healthcare professionals (25.71%), and 8 were researchers (22.86%). Two participants identified themselves as both a patient and a healthcare professional, and one participant identified as a patient and researcher, however, these professional roles were unrelated to retinoblastoma. Patients were classified according to the SPOR definition [6], including patients, family members, and informal caregivers. CRRAB members most commonly learned about the second annual CRRAB meeting also from a member of the research team that initiated CRRAB (22/35, 62.86%). Participants also learned about CRRAB from a healthcare professional (6/35, 17.14%) and social media, emails or pamphlets (5/35, 14.29%). A minority of participants (2/35, 5.71%) learned about CRRAB from a family member or patient.

Information was collected on previous involvement with CRRAB, prior to the second annual general CRRAB meeting. The largest number of participants were not previously involved (14/35, 40%), while 28.57% of participants (10/35) were involved for 9–12 months, since the inception of the Canadian Retinoblastoma Research Advisory Board. The rest of participants were involved for a range of months (11.43%; 4/35 for 3–6 months, 8.57%; 3/35 for 6–9 months, and 5.71%; 2/35 for less than 1 month and 1–3 months). Ten participants (28.57%) attended the first annual CRRAB meeting, and 17 participants (48.57%) were registered to attend the second annual CRRAB meeting in 2017.

### Evaluation of CRRAB

#### First annual CRRAB meeting

##### Pre- and post-meeting questionnaire (Table 2)

The mean changes in pre-test and post-test questionnaires are presented in Table 2 with the mean difference. There were shifts in attitudes towards the expected responses in five out of the six statements (statements 1, 2, and 4–6). In general, most members agreed that research is relevant to all stakeholders in the scientific community and that patients can have important impacts on research. Overall the results were not significant (*p* = 0.625), and there was very little change between the pre- and post-test questionnaire. Twenty-two individuals completed the pre-test questionnaire including demographics, while 20 completed the post-test questionnaire.

**Table 2:** Mean changes in questionnaire responses for pre- and post-test and statistical summary of the paired t-test

Question	Pre-Test Mean (*n* = 20)	Post-Test Mean (*n* = 20)	Difference Mean
1. Retinoblastoma research is only relevant to clinicians.	4.71	4.86	0.14
2. Retinoblastoma clinicians lack the knowledge or skills needed to use research in their practice.	4.14	4.33	0.19
3. Retinoblastoma research is not relevant to the day-to-day lives of patients.	4.67	4.67	0.00
4. All patients should be given the opportunity to learn about, and participate in, retinoblastoma research.	1.48	1.29	0.19
5. Patients are encouraged to be involved in retinoblastoma research.	1.76	1.67	0.10
6. I can have a meaningful impact on retinoblastoma research.	1.62	1.33	0.29
Statistical Summary			
Mean	3.06	3.02	
Standard Deviation	1.60	1.76	
Standard Error Mean	0.65	0.72	
N	6	6	

*P*-value = 0.625, M = 0.0397, SD = 0.1867, 95% CI [−0.1562, 0.2356].

##### Chart board discussions (Table 3)

Three themes emerged when discussing participant’s goals related to CRRAB involvement. First, “knowledge of RB” (10 references, 55.55%), suggested there was room to both improve learning for participants and education of the public and medical community about retinoblastoma. Specifically, participants felt they could improve their own knowledge of the latest research as well as learn from other patients themselves. There was a suggestion also to improve knowledge of retinoblastoma among medical students and improve increased public awareness of retinoblastoma... Second, the theme of “innovation” (6 references, 33.33%), centred on generating novel research ideas through research-patient partnerhsips, improving patient care, and increasing awareness and education among patients to self-advocate about their follow-up care. Third, the theme of “patient-oriented care and research” (6 references, 33.33%), delved into increasing support for survivors and families, advocacy for patient involvement in research, and facilitating patient engagement in research.

**Table 3:** Qualitative analysis of Chart Board Questions 1–3 at CRRAB 2016 including themes, number of references, answers and coverage

Theme/Node	References (n, %)	Answers
Question 1: By participating in the CRRAB I want to…		
Knowledge of RB	10, 55.55%	- Learn from patients.
		- Learn about all the latest research.
		- Get the word out there in public
		- Educate all oncologists and continue to do so about the secondary risks and monitoring for all RBs throughout lifetime.^a^
		- Communicate risks and monitoring of secondary cancers for all Rbs
		- Inform future patients to better understand Rb.
		- Develop ideas for increased awareness in Rb.^a^
		- Tell/inform as many healthcare providers about this project; tell Rb families
		- Communicate post-Rb secondary health conditions; not cancer, re: immunity.
		- Target all Canadian medical schools to educate student doctors about monitoring for secondary cancers for all Rbs throughout lifetime.^a^
Innovation	6, 33.33%	- Improve global outcomes for Rb families.
		- Be in a position to effect change
		- Generate a unique researcher-patient model for producing research ideas and projects.
		- Generate novel ideas for research in Rb and associated diseases
		- Develop ideas for increased awareness in Rb.^a^
		- Develop a handbook for 18+ individuals affected by Rb, which would also include a self-advocacy guide in how to request monitoring or tests for possible secondary masses to be looked into that could bridge the language between doctor and patient to avoid miscommunication or bridges being burnt.
Patient Oriented Care and Research	6, 33.33%	- Support survivors and families
		- Incorporate patient perspective into research/study design to maximize the study potential.
		- Learn from patients.
		- Educate all oncologists and continue to do so about the secondary risks and monitoring for all Rbs throughout lifetime.^a^
		- Better advocate for involvement of families and patients in research.
		- Target all Canadian medical schools to educate student doctors about monitoring for secondary cancers for all Rbs throughout lifetime.^a^
Question 2: What questions about retinoblastoma would you like to see answered by research?		
Bio-medical Focused	9, 64.29%	- How to prevent retinoblastoma?
		- Early detection?
		- What is a “bad orbital ray”?
		- Whole eye transplant?
		- IVF and Rb?
		- Blood cord?
		- What are the second malignancy issues facing Rb survivors and how can we explore better?
		- Common threads between different mutations?
		- Differences in Unilateral, Bilateral, and Mosaicism – what do these mean?
Patient Focused	5, 35.71%	- Importance of family communication and telling risk to family members?
		- What are the major worries and concerns facing Rb survivors?
		- How can we improve GP and pediatrician training in recognizing and referring Rb?
		- How can we better support Rb patients during Rb?
		- What are the long-term issues facing Rb survivors?
Question 3: How can we better engage patients and families in retinoblastoma research?		
Community Outreach	5, 41.67%	- Use social media
		- Send quarterly (or 2x / year), plain language summaries of Rb research in Canada; “Rb Newsletter”.
		- Inform clinics that do flu shots that this is an option (+ genetic counselors, Ochealth, etc.).
		- Create social media support group
		- Social media blog.
Personalized Contact	4, 33.33%	- Having multiple modes of communication for families
		- Identify Rb patients
		- Approach Rb patients who are mature enough to participate and be positively engaged.
		- How can we *carefully engage patients? Risk?
Education	3, 25.00%	- Understanding the current standard of care.
		- Ensuring a mutual comprehension; i.e. training.
		- Demonstrate the power of research to understand the value “time well spent”.

^*^statement was classified under more than one theme.

Questions about retinoblastoma that participants wished to see answered were mainly bio-medical focused (64.29%; 9/14), covering disease prevention, early detection, examining genetic variants and phenotype. A smaller proportion of questions were patient-focused (5.71%; 5/14) and directly related to the patient experience, including patient-physician interaction, psychosocial support of fmailies, and survivorship.

Three themes emerged when discussing methods to better engage patients: community outreach (5/12, 41.67%), for example through social media and newsletters; direct communication/personalized contact (4/12, 33.33%), with an emphasis of protecting the patient and being ‘careful’ around research engagement; and, education (3/12, 25.00%), suggesting that effective patient engagement in research follows sufficient knowledge of RB care and research.

### Second annual CRRAB meeting

#### Pre-meeting questionnaire

**Fig. 1:**
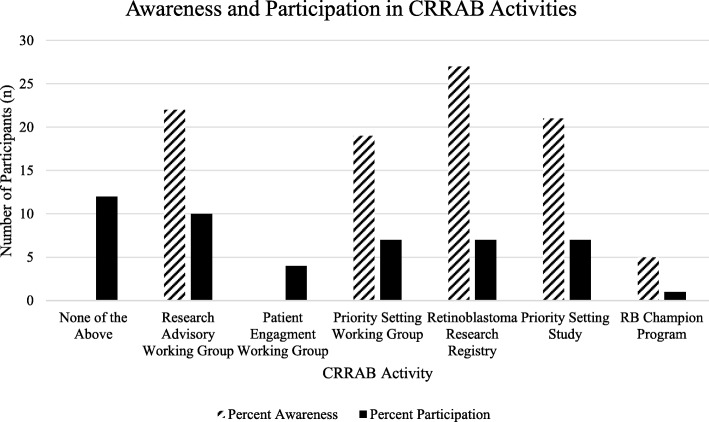
Awareness of and participation in CRRAB groups and activities

One year following the creation of CRRAB, 94.3% of participants were aware of at least one CRRAB activity, while 65.7% had participated in one in the last year (Fig. 1). The majority of participants were aware of the Retinoblastoma Research Registry (27/35, 77.14%), however, just 7 participants (7/35, 20.00%) out of the 21 eligible patients reported having participated in it. Most participants were aware of the CRRAB working groups, specifically the Research Advisory, Patient Engagement and Priority Setting working groups (22/35, 62.86%; 17/35, 48.57%; 19/35, 54.29%, respectively). Fewer participants reported participating in these groups (10/35, 28.57%; 4/35, 11.43%; 7/35, 20.00%, respectively). The majority of participants were aware of the priority setting study that was occurring at the time of the survey (21/35, 60.00%) yet only 20.00% participated (7/35). 14.29% (5/35) participants were aware of the RB Champion program, while only 1 participant (1/35, 2.86%) took part in it.

**Fig. 2:**
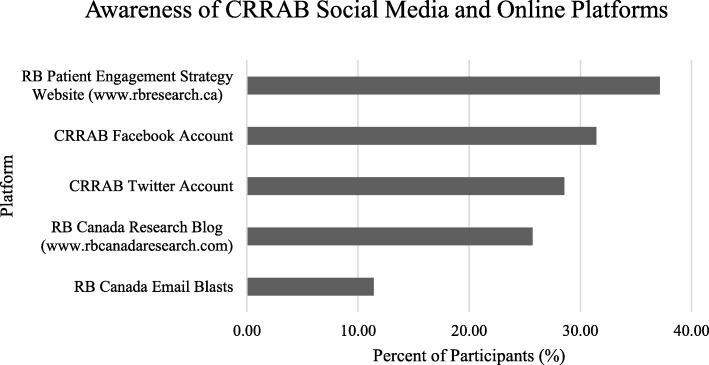
Participant awareness of social media and online platforms

Less than half of participants were aware of the various forms of social media and online engagement (Fig. 2). 37.14% of participants (13/35) were aware of the Patient Engagement Strategy website, 31.43% (11/35) and 28.57% (10/35) of participants were aware of the CRRAB Facebook and Twitter accounts, respectively. Nine participants (25.71%) were aware of the RB Canada Research Blog and four participants (11.43%) were aware of the RB Canada Email Blasts. Participants were also asked how often they engaged with the same social media platforms. The mean scores for engagement with all platforms was 2.00 (rarely) and below (Table 4).

**Table 4:** Mean scores, median, mode and range for engagement with CRRAB social media and online platforms

On a scale of 1–5 (1 - never, 5 - often), how often did you read/engage with:	Mean	Median	Mode	Range
RB Patient Engagement Strategy Website (www.rbresearch.ca)	1.77	1.00	1.00	1–4
RB Canada Research Blog (www.rbcanadaresearch.com)	1.80	1.00	1.00	1–5
RB Canada Research Email Blast	2.00	1.00	1.00	1–5
CRRAB Twitter Account (https://twitter.com/rb_research)	1.83	1.00	1.00	1–5
CRRAB Facebook Account (https://www.facebook.com/RBresearch/)	1.80	1.00	1.00	1–5

1 = never, 2 = rarely, 3 = neutral, 4 = sometimes, 5 = often

35) were involved/35) were involved in some other form of patient engagement, with the distribution shown in Table 5.

**Table 5:** Participant involvement in other patient engagement activities

Beyond CRRAB, have you participated in any other patient engagement activities in 2017?	N	Percent (%)
I participated in a research study as a study subject.	7	20.00
I was a team member on a research study.	12	34.29
I was a team member on a grant application.	9	25.71
I contributed to writing a scientific article.	10	28.57
I contributed to writing a summary of scientific research.	7	20.00
I attended an information session/workshop about patient engagement in research.	10	28.57
I attended a conference with strong patient participation and inclusion.	7	20.00
Total Engaged (across all other activities)	24	68.57

Overall participants agreed that CRRAB made research more accessible, encouraged involvement and provided opportunities (Mean Scores: 4.06, 4.31, 4.40) (Table 6). The statements regarding the diversity of retinoblastoma clinicians and researchers engaged with CRRAB had mean scores of 3.54 and 3.71 respectively. Lastly, there was agreement that patient engagement in research will have a meaningful impact on retinoblastoma research (Mean Score: 4.40).

**Table 6:** Agreement with statements regarding CRRAB and patient engagement

On a scale of 1–5 (1 - strongly disagree, 5 -strongly agree), how would you rate the following statements?	Mean	Median	Mode	Range
CRRAB engages a diverse group of retinoblastoma clinicians.	3.54	3	3	2–5
CRRAB engages a diverse group of retinoblastoma researchers.	3.71	3	3	2–5
CRRAB contributes to making research accessible to retinoblastoma survivors and family members.	4.06	4	4	2–5
CRRAB encourages retinoblastoma survivors and family members to be involved in retinoblastoma research.	4.31	4	5	3–5
CRRAB provides opportunities to retinoblastoma survivors and family members to be involved in retinoblastoma research.	4.40	5	5	3–5
Patient engagement in research will have a meaningful impact on retinoblastoma research.	4.40	5	5	3–5

1 = strongly disagree, 2 = disagree, 3 = neutral, 4 = agree, 5 = strongly agree

#### Chart board discussions (Table 7)

Participants proposed ideas for future CRRAB goals and activities, and results fell into 6 main themes. First, the theme of ‘advocacy and education’ (16/40, 40.00%) centred on raising awareness on RB as well as on CRRAB activities. Second, the theme of ‘increase engagement’ (8/40, 20.00%) included comments on raising the number of families involved, and to specifically involve clinicians and researchers with specific expertise (i.e. second cancers). Third, the theme of ‘innovation’ (8/40, 20.00%) suggested that CRRAB should rally around specific unanswered research questions and solved them, together with patient support. Fourth, the theme of ‘collaboration’ (7/40, 17.50%) centred on identifying and making strategic partnerships with individuals who could advance research goals. Fifth, the theme of ‘refine goals’ (6/40, 15.00%) suggested a process was needed in order to reach future goals and activities. Finally, ‘psychosocial support’ (4/40, 10.00%) was identified as a goal of CRRAB, through the development of support groups, social events and tangible resources.

**Table 7:** Qualitative analysis of Chart Board Questions 1–5 at CRRAB 2018 including themes, number of references, answers and coverage

Theme/Node	References (n, %)	Answers
Question 1: In 2018 CRRAB Should		
Advocacy and Education	16, 40.00%	- Clinicians should present debates about different treatment options
		- Make content more engaging and less dry
		- Walk in the santa clause parade
		- Create posters for health care settings
		- Link resources on the website
		- CRRAB involved in schools?
		- Develop pamphlet to hand out… at time of diagnosis or 1 year later
		- Rb specific awareness campaign
		- Awareness campaign
		- WECH – International RB week
		- To become a non for profit independent organization
		- Raise public awareness of retinoblastoma through a dedicated week, nationally across Canada, include politicians
		- For young survivors develop simple catchy RB (songs/videos/cartoons) education on some key topics or questions that they have
		- Digital stories of survivors and families
		- Update social media e.g., summary video, to explain what CRRAB accomplished
		- Communication plan
Increase Engagement	8, 20.00%	- Double (at least) the number of families connected
		- Social event in September (childhood cancer awareness month)
		- Become a NFP (model)**
		- In 2018 CRRAB should be continuing outreach to recruit more patient and family involvement as well as researchers involvement/ recruitment**
		- Identify key clinicians and researchers in each province to learn about and potentially collaborate with CRRAB**
		- More broad family involvement
		- Communication plan
		- Engage clinicians and researchers from the sarcoma with regard to second cancers
Innovation	8, 20.00%	- Prioritize a specific research project**
		- Focus on the top 3 questions**
		- Lobby support for DePICT project
		- Develop pamphlet to hand out… at time of diagnosis or 1 year later**
		- Long-term effects
		- Focus on palliative care for retinoblastoma
		- Focus on treating and reducing chronic symptoms/side effects of retinoblastoma (like dry eyes, inflammation)
		- Start mobilizing research and patient community around top 10 priorities
Collaboration	7, 17.50%	- Connect to other global RB research groups
		- Become a NFP (model)**
		- In 2018 CRRAB should be continuing outreach to recruit more patient and family involvement as well as researchers involvement/ recruitment**
		- Identify key clinicians and researchers in each province to learn about and potentially collaborate with CRRAB**
		- Palliative care – Canada and international
		- Engage clinicians and researchers from the sarcoma with regard to second cancers**
		- Include more researchers from graduate programs and universities
Refine Goals	6, 15.00%	- Prioritize a specific research project**
		- Focus on the top 3 questions**
		- Clarify time commitment for working groups
		- Define projects
		- Establish/ define primary research projects
		- Focus on another “3 goals” to consolidate group and experience success
Psychosocial Support	4, 10.00%	- A social event to facilitate engagement
		- Peer support groups
		- Support group for recent diagnosis
		- Develop pamphlet to hand out… at time of diagnosis or 1 year later**
Question 2: To have more patient and family leadership in CRRAB we should…		
Increase Access	6, 42.86%	- Have a coordinated schedule for committee meetings and maybe have webinar meetings
		- Facilitate telecom/travel/regional work
		- Send more opportunities via email
		- Paid position to facilitate and feedback
		- Schedule non-in person
		- Multiple ways to input
Partnership	5, 35.71%	- Explore co-leadership opportunities
		- Offer a subcommittee for both patient and then family subcommittee
		- Help patients and families understand what their role would be and why it’s important
		- Paid position to facilitate and feedback
		- Support
Outreach	5, 35.71%	- Ask them
		- Send more opportunities via email
		- Continue to get the word out
		- Ask
		- Hold social events
Innovation	4, 28.57%	- To foster new research ideas/ collaborations
		- To develop eye care screening for infants, and research on possible aftercare streamlining on RB and other diseases
		- Facilitate research
		- Inform research and engage patients with it
Question 3: I want to be part of CRRAB working groups because…		
Benefit RB Families	5, 38.46%	- To add value to a specific cause
		- To provide a tangible benefit
		- To comfort others**
		- We can make a difference in our children’s lives and those not yet diagnosed with RB
		- I want my daughter to learn/see/know that we can conquer RB and live a meaningful full life
Community	4, 30.77%	- To fill gaps in CRRAB
		- To comfort others**
		- To stay connected with other members of the RB community.
		- We’re stronger together
Include Patients	3, 23.08%	- Patient/family oriented research is an important emerging perspective.
		- This gives patient focus to our work
		- I want to be involved because patient involvement is the new impetus for patient need added to theory
Leadership	2, 15.38%	- I would like part of Steering and Business development committee. I would take part as a leader.
		- To keep involved in this important initiative
Question 4: I don’t want to be part of CRRAB working groups because…		
Time and Cost Restraints	7, 36.84%	- Time commitment
		- Scheduling
		- Overnight flights and non funded time is tough
		- Life is busy
		- (I actually do) but lack of time to share across commitments is what prevents more involvement
		- Time
		- Afraid of commitment
Burnout	3, 15.79%	- Maybe people don’t understand what it involves or has had research participant burnout or never getting post research feedback**
		- Feedback of research result “used”
		- Burnout
Lack of Understanding	3, 15.79%	- Maybe people don’t understand what it involves or has had research participant burnout or never getting post research feedback**
		- Awareness
		- Communication plan
Psychosocial Issues	2, 10.53%	- Self esteem
		- Provide support – painful memories/ active disease treatment
Conflict of Interest	1, 5.23%	- Ethical issues or conflict of interest
Question 5: The purpose of CRRAB is to…		
Increase Collaboration	10, 37.04%	- RB intersect point
		- Bring together patients, clinicians, researchers to initiate dialogue
		- To foster new research ideas/ collaborations
		- Push for research and implementation of a collaborative health care network for RB across Canada**
		- Linking/ integrating efforts
		- Co-investigators – patients, families**
		- Ideas outside our own box
		- Collaborations
		- Solidify the RB group
		- Concerted effort to bring patients and professionals together
Community	6, 22.22%	- RB survivors network
		- Connect other families
		- Provide a social and informative environment**
		- Concerted effort to bring patients and professionals together
		- Create a community
		- Networking opportunities
Improve Care	5, 18.51%	- Diagnosis - > death … whole span of care
		- Push for research and implementation of a collaborative health care network for RB across Canada**
		- To develop eye care screening for infants, and research on possible aftercare streamlining on RB and other diseases
		- Translate to include Child’s Life daily
		- Help standardize practices
Engage Patients	4, 14.81%	- To setup a Canadian research team with patient focus involvement
		- Include patients/families in every stage of research and dissemination (Co-Investigators)
		- Co-investigators – patients, families**
		- Inform research and engage patients with it
Awareness and Advocacy	4, 14.81%	- Raise awareness and showcase outside RB specific community
		- Promote Education of RB
		- Bring awareness to other medical professionals
		- Provide a social and informative environment**

**statement was classified under more than one theme

Participants’ ideas of how to increase patient and family leadership revealed four themes: ‘increase access’ (6/14, 42.86%), for example, through routine communications via email and teleconference, and compensation of participants; ‘partnership’ (5/14, 35.71%), by offering paid leadership positions and defining speficic roles; ‘outreach’ (5/14, 35.71%), by proactively reaching out to patient and families through social events and telecommunications; and ‘innovation’ (4/14, 28.57%), that is, to facilitate research ideas that engages patients.

Participants wanted to be involved to benefit the RB community (5/13, 35.71%), be part of a community (4/13, 30.77%), include patients (3/13, 23.80%) and be leaders (2/13, 15.38%). Participants did not want to be involved in working groups due to time and cost restraints (7/19, 36.84%), burnout (3/19, 15.79%), lack of understanding (3/19, 15.79%), psychosocial issues (2/19, 10.53%) and/or conflicts of interest (1/19, 5.23%). 21.05% of responses (4/19) were excluded because they didn’t contribute meaningfully to a theme.

Participants also shared their thoughts on the purpose of CRRAB, and results were classified under five themes: increase collaboration (10/27, 37.04%), community (6/27, 22.22%), improve care (5/27, 18.51%), engage patients (4/27, 14.81%), and awareness and advocacy (4/27, 14.81%). One response (3.70%) was excluded because it did not contribute meaningfully to a theme.

#### Post-meeting questionnaire

**Fig. 3:**
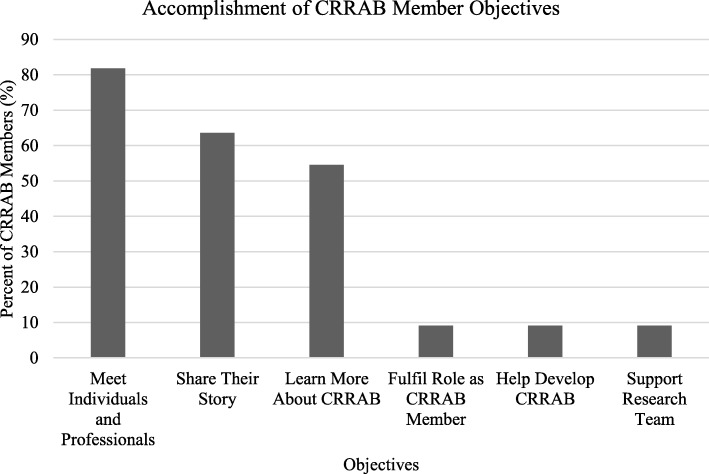
CRRAB member objectives at second annual general meeting

Eleven participants completed the post-meeting questionnaire. Nine (81.81%) and seven (63.63%) respondents accomplished their objectives of meeting individuals and professionals, respectively (Fig. 3). Five respondents (45.45%) accomplished their objective of sharing their story, while six (54.54%) accomplished their objective of learning more about CRRAB. The remaining respondents indicated that the four objective statements did not apply to them. Others responded that their objectives included fulfilling their role as CRRAB member (1/11), helping develop CRRAB (1/11), and supporting the research team (1/11). The majority of CRRAB members (90.9%; 10/11) felt that the next steps for CRRAB were clear while all participants signed up to participate in a CRRAB working group.

### Impacts, context of PPI

Given the genetic and long-term implications of a retinoblastoma diagnosis, patients are incentivized to contribute to and help co-create research. CRRAB is a means to reach and sustainably engage a diverse group of retinoblastoma patients consistently and appropriately. Though it is difficult to assess the impacts of PPI so early in CRRAB’s history, preliminary results are promising. Our results showed significant overall patient awareness of CRRAB activities (Figs. 1 and 2) and 66% of participants reported participating in at least one CRRAB activity (Fig. 1). The development and evolution of CRRAB involved patients throughout the process, and this study is a means to uncover and incorporate patient feedback into its operations.

## Discussion

### Summary of main conclusions

CRRAB aims to connect patients to retinoblastoma research, engage a diverse group of patients, and support and create co-directed retinoblastoma research. Participants in this study supported the ideals of patient-oriented research before the first CRRAB meeting and continued to support these through the second annual meeting’s goal setting exercises and group discussions. The majority of participants were aware of ongoing CRRAB activities, but a subset (34%*) revealed they had not participated in activities beyond the annual general meeting.

### Importance of engagement for the RB community

A diagnosis of retinoblastoma has a substantial life-long impact on not only the individual with the diagnosis, but also on caregivers and family members. Since retinoblastoma patients often have extended relationships with the healthcare and research communities, there is a unique opportunity to co-create research and share knowledge. Patients and their families gain unique expertise and knowledge of retinoblastoma based on their personal experience with their illness. This includes preferences, attitudes towards risk, values, habits and behaviours [17]. This wider perspective can improve the quality - and, in turn, impact - of research by making it more meaningful and relevant [18]. Engaging patients in all aspects of research and in authentic partnerships based on mutual respect and shared decision making promotes patient leadership [17]. Indeed, engaging patients as equal partners removes the former hierarchy that could impede authentic patient engagement [17].

The importance of patient oriented research amongst the retinoblastoma community was apparent before the first CRRAB meeting (Table 2). At the second CRRAB meeting, participants cited the desire to be a leader as one of their motivations to be part of CRRAB, further supporting the importance of patient engagement in research. Participants also suggested ideas to increase patient and family leadership, with common themes featuring increasing access, creating partnerships, and improving outreach and innovation. CRRAB members had strong agreement that CRRAB makes research more accessible, encourages involvement and provides opportunities (Table 6). CRRAB is co-creating research with retinoblastoma patients involved at each step of the process, including the creation of the advisory board itself. By seeking patients’ thoughts and opinions and incorporating shared decision making, CRRAB facilitates patient engagement and shifts the focus from “research for patients” to “research with patients”. CRRAB continues to implement new methods to evolve the national patient engagement strategy, sustain meaningful partnerships and promote patient leadership.

### Methods of engagement

In a systematic review, the use of an advisory board was described as the most active form of patient engagement in research [19]. Patient engagement exists along a continuum [20] and CRRAB was established with the goal of engaging patients at across all areas of the research process. The second annual CRRAB meeting was held in conjunction with a national research priority setting exercise. In accordance with the idea of a continuum of engagement, patients could define their level of participation in setting retinoblastoma research priorities. Patients had the opportunity to suggest research questions, participate in the ranking exercise, attend the annual CRRAB meeting, join the working group meetings, and/or sit on the steering committee.

Swartz et al. successfully engaged patients and stakeholders as partners in research by co-developing a study protocol and jointly executing a randomized controlled trial [21]. Similarly, the CRRAB Research Development working group is actively collaborating to develop a study protocol and apply for funding to answer one of the “top 10” retinoblastoma research priorities. Other studies also described successful patient engagement through regular meetings to provide input and make decisions regarding study progress [22].

At both the first and second CRRAB meetings, word of mouth or personal invitations were the strongest predictors of involvement. Not surprisingly then, suggested methods to better engage patients included community outreach, direct communication, personalized contact, and improving patient education (Tables 3 and 7). This also suggests that increased effort is necessary towards identifying target individuals who are currently outside the CRRAB network, so that personal invitations can be made. The aim of the RB champions program, a group of individuals across Canada who share their personal stories and promote CRRAB initiatives [10], is to address this need and engage more diverse set of retinoblastoma patients.

### Population characteristics as measure of engagement

The majority of participants who completed the surveys at the first (50%) and before the second (60%) annual CRRAB meeting (Table 1) were retinoblastoma survivors or parents of children diagnosed with retinoblastoma. This met the initial aim of engaging 50% or greater patients within CRRAB, a measure that showed strong patient involvement. We felt it was important that CRRAB was composed of 50% or greater patients to ensure equal balance of power between patients, healthcare professionals, and researchers. This goal was also in line with fostering an equal partnership, a critical standard for successful patient engagement [17]. The increased proportion of patients also demonstrates increased reach and growth between CRRAB meetings as the number of patients nearly doubled (from 11 to 21), while the numbers of healthcare professionals and researchers only increased only marginally (Table 1).

CRRAB meeting attendees were largely older survivors or recently affected families. Although only empirically collected for the first meeting (Table 1), this finding was anecdotally observed at the second meeting as well. This suggests the need to improve our engagement techniques to better involve teenagers and young adult survivors. The lack of young adult survivors might be due to the nature of retinoblastoma affecting infants, which means survivors often have no memory of the experience or treatment. As well, it could potentially be a result of recruitment methods, suggesting a need to determine how best to recruit young adults. Lastly, it is important to consider the psychosocial impact of participating in research. Concerns have been raised about individuals re-living what might be considered a negative experience by participating in research. However, patients with a cancer experience have described research involvement as therapeutic [23]. Prior research indicates that parents may struggle with maladaptive coping mechanisms around the time of retinoblastoma diagnosis [2], thus these individuals may be less likely to participate in CRRAB and its activities. We are increasing efforts to reach these uninvolved groups by introducing other forms of social media such as Instagram (@rb_research) and posting regularly to further our reach and provide information to all eligible individuals. Valerio et al. compared two sampling methods to engage hard-to-reach communities in research priority setting and determined that snowball sampling or using a chain-referral method to recruit patients was effective. We have attempted to implement a similar recruitment method by introducing RB champions to reach uninvolved individuals [24].

At the second annual CRRAB meeting, most participants were not previously involved in CRRAB, which demonstrates large growth within the year-long existence of the advisory board. Ten individuals who completed the second annual CRRAB meeting survey attended the first CRRAB meeting and had been involved since the inception of CRRAB. Although this might indicate decreased involvement from the remaining 12 individuals at the initial CRRAB meeting, this is likely a result of scheduling conflicts.

### Motivations and benefits of engagement

Participants cited numerous reasons why they were motivated to engage in research (Tables 3 and 7). The most common motivations - improving knowledge of RB and educating others - reflects the idea of patient engagement as a method to share knowledge and learn from others’ expertise. While engaging with CRRAB provides an opportunity to share experiences in order to help others, it also provides an opportunity to learn from experts in the field as equal partners. This was also supported by the theme surrounding community, both benefiting from and being part of the RB community. The theme of contributing to innovation supports the notion of sharing the patient perspective to enhance research but takes it one step further to being creators of novel research. In line with one of the goals of patient engagement, this would hopefully enhance the uptake of results [4]. Patients were also motivated to be part of CRRAB to focus research on the patient and align research goals with their own. This is why conducting a priority setting exercise, which was one of the early goals of CRRAB, was important to identify diverse perspectives and jointly determine research priorities. All of the motivation themes that arose are in line with the SPOR framework for patient-oriented research [5], suggesting that the retinoblastoma community believes in the ideals of patient engagement, supporting the need for patient engagement in the retinoblastoma community. The themes also suggest benefits of patient engagement, including ones not previously considered, such as the opportunity to be a leader. This shows that patients want to lead research and share their expertise. This opportunity is offered to patients with CRRAB, allowing members to engage how they prefer, from being registry members to taking on elected leadership positions.

### Barriers to patient engagement

The most common barriers to participating in CRRAB working groups cited by participants are related to time and cost constraints, both of which coincide with barriers previously suggested by literature [19]. We address these barriers by surveying all participants for best meeting times, conducting meetings online, allowing patients to determine how much time they provide, and providing travel stipends to attend in-person meetings. As well, the theme of “lack of understanding” aligns with the concern that patients may include research questions that are unfeasible [19]. We help address this barrier by supporting patients and encouraging active engagement. The conflict of interest category represented one response, however the source is unknown. Potentially, a clinician might feel a conflict of interest when working closely with a parent as they should feel leadership over clinical care. A previously suggested barrier of research becoming tokenistic and devaluing the patient’s input [25, 26] did not arise, indicating that CRRAB is a true and valuable form of patient engagement, and an opportunity for patients to contribute meaningfully. We are attempting to eliminate time and financial barriers with the introduction of a new role within the retinoblastoma research team. A grant was obtained to hire a full-time research coordinator with lived experience of retinoblastoma (patient or family member).

### Awareness and engagement in activities

Figures 1 and 2 and Table 4 describe percent awareness of and engagement in CRRAB activities and online platforms. Despite the research registry requiring minimal participation (completing registration with no further commitments), participation was surprisingly low. This result was interesting and should prompt further investigation into reasons for not participating. Patients might be concerned about being asked to be part of studies or receiving too many emails. We hope to mitigate this concern by educating the retinoblastoma community about the registry and its requirements, indicating that patients can engage with it however they choose. The lower awareness of and engagement with online platforms (Fig. 2 and Table 4) compared to in-person initiatives may be a result of the demographics of participants. Studies suggest that older individuals may be less likely to engage with online social media platforms [27], however we did not collect age with the demographics questionnaire. As well, patients with visual impairments may face difficulty engaging with online social media. For this purpose, we aim to design all our tools to be accessible. The lower levels of engagement compared to awareness may represent the previously discussed barriers to participation. The large awareness of CRRAB initiatives demonstrates the numerous goals CRRAB has accomplished within a short time period, many of which have been largely driven by patients.

While the majority of CRRAB members reported being engaged in CRRAB activities, a significant proportion (34%) of the membership were passive participants, attending meetings but not participating in additional CRRAB activities (e.g. developing research proposals, becoming RB Champions). This might mean that CRRAB membership represents a natural distribution of involvement from passive to active, including individuals who are typically difficult to involve in research activities. Although an advisory board is considered one of the most active forms of patient engagement [19], CRRAB has been designed so that individuals can choose to participate each according to their interests, skills and availability. The involvement of both passive and active participants in CRRAB suggests that overall leadership and decision-making is shared and does not reflect only one type of participant. A future direction is to further understand the motivations and interests of this passive subgroup, and identify if there are other ways to increase their engagement, or if they desire to remain as they are.

### Perception of CRRAB impact and environment

Mean agreement scores (Table 6) supported the idea that CRRAB made research more accessible, encouraged involvement and provided opportunities for involvement. This agreement suggests that CRRAB is a valuable and effective framework for improving patient engagement. Although mean scores were towards agreement, participants felt less confident in the diversity of clinicians and researchers engaged in CRRAB. This is likely because the retinoblastoma community in Canada is small and individuals often already know one another.

### Perceptions of and agreement with CRRAB mandate

At the end of the second annual CRRAB meeting, participants were asked what they thought was the purpose of CRRAB (Table 7). Analysis revealed themes of increasing collaboration, creating or contributing to a community, improving care, engaging patients, and improving awareness and advocacy. Similar to participants’ personal goals, these themes support the SPOR framework [5], indicating that participants understand and believe in the ideals of patient engagement in research. In the post-meeting questionnaire, all participants that had set objectives felt that they had fulfilled them, supporting the importance of an annual in-person meeting (Fig. 3).

Participants shared motivations and reasons for joining CRRAB and indicated what they wanted to accomplish at the first CRRAB meeting (Table 3). Comparing this result with involvement in CRRAB initiatives at the second meeting, we can see that CRRAB members accomplished many of their goals during the year. CRRAB members wanted to share knowledge of RB, which was done through social media, email blasts, and blog posts. Participants wanted to contribute to innovation in research, with an example being to “generate a unique researcher-patient model for producing research ideas and projects”. This was accomplished with the development of the CRRAB framework and the process to determine the “top 10” retinoblastoma research priorities (publication pending). Lastly participants wanted to increase and improve patient-oriented research, and as mentioned this is a fundamental goal of CRRAB and was considered in the development of all CRRAB initiatives.

### Limitations

It is important to acknowledge potential limitations of this study. With respect to recruitment and population, although the study was Canada-wide and open to individuals of varying ages and experiences with retinoblastoma, the majority of participants were parents from Ontario, followed by adult survivors. We acknowledge difficulty in recruiting and reaching individuals affected by retinoblastoma as a result of the nature of the disease; individuals are affected at a young age, and as such their parents are typically the ones engaging with the health and research landscape. The CRRAB Patient Engagement working group is working towards expanding nation-wide involvement of survivors and expanding opportunities for adolescents and young adults to participate. The study also only reflects the population who was able to attend the annual general meeting, and may have excluded patients who participated in other CRRAB activities but missed the meetings.

With respect to limitations in methodology, the second annual CRRAB meeting was held in conjunction with a priority setting workshop. Those that participated and discussed research priorities might have been influenced by their experience in the workshop. Information on retinoblastoma diagnosis was not collected at the second annual CRRAB meeting which limits our ability to analyze changes in demographics of retinoblastoma patients. This would have potentially allowed us to see if CRRAB was involving more young adult survivors. It is important to note that we did not distinguish between types of participant for the chart board discussions, as such it is not known whether suggestions came from patients, healthcare professionals, or researchers. The change in survey method between the first and second meeting limited comparison between years, however we believe this strengthened the quality of information obtained at the second meeting.

Lastly, CRRAB is currently being driven by researcher involvement and a lack of self-sustaining driving force may be a limitation for its longevity.

### Current and future directions

CRRAB continues to evolve as a framework for patient engagement and actively works to eliminate barriers to effective engagement. CRRAB has engaged more patients, increased the number of initiatives and successfully completed multiple projects. This study adds important knowledge on the practice of patient engagement in action and shares lessons learned and directions for future research. The third annual general CRRAB meeting was successfully held in early 2019 in conjunction with a retinoblastoma family gathering and a “Top 10” Priorities dissemination activity. An individual with a lived experience of retinoblastoma was hired as a research coordinator, with the title Parent in Research (IR) and is leading several projects, including leading the Research Development Working Group to uncover and address the psychosocial needs of retinoblastoma patients and their families. This, and all other patient engagement activities are being evaluated using the Patient and Public Engagement Evaluation Tool (PPEET) [28]. This supports the need for future studies to use predefined, validated tools to evaluate patient engagement at regular intervals [29]. In future, we will explore the analytics behind CRRAB social media reach and use to help clarify the effects of these awareness and recruitment measures. CRRAB will continue to evolve and address its goals, including the themes presented at the second annual CRRAB meeting such as advocacy and education, increasing engagement, innovation, collaboration, refining goals and providing psychosocial support. Although CRRAB is currently researcher-initiated and patient-driven, the eventual goal is to create a self-sustaining advisory board with all organizational and leadership roles held by patients.

## Conclusion

The results of this study suggest that CRRAB supports engagement of patients in retinoblastoma research in ways that are amenable to patients, researchers and healthcare professionals. The advisory board has helped create meaningful co-directed research that has engaged patients throughout the entire process. CRRAB members supported the ideals of patient engagement in research and suggested methods to eliminate barriers to patient engagement. Involving patients in each step of the process enabled CRRAB to eliminate barriers and engage a diverse group of patients, making CRRAB a strong example of patient engagement in research. CRRAB will continue to be used as a framework for patient engagement, with improvements based on participant feedback. Future studies will evaluate CRRAB using PPEET as a validated survey tool.

## Supplementary information

**Additional file 1:** GRIPP-2 Reporting Checklist.

**Additional file 2:** First Annual CRRAB Meeting Pre-and Post-Test Questionniare.

**Additional file 3:** Second Annual CRRAB Meeting Evaluation.

## Data Availability

The datasets used and/or analysed during the current study are available from the corresponding author on reasonable request.

## Abbreviations

CRRAB: Canadian Retinoblastoma Research Advisory Board
GRIPP-2: Guidance for Reporting Involvement of Patients and the Public 2
PPEET: Patient and Public Engagement Evaluation Tool
PPI: Patient and Public Involvement
RB: Retinoblastoma
SPOR: Strategy for Patient-Oriented Research
WG: Working Group
