# Supplementary material for: The Canadian retinoblastoma research advisory board: a framework for patient engagement
Source: Res Involv Engagem. 2020 Feb 28;6:7. doi: 10.1186/s40900-020-0177-8 (PMC7048037; doi:10.1186/s40900-020-0177-8)
Supplement: Supplementary file 2 — Additional file 2: First Annual CRRAB Meeting Pre-and Post-Test Questionniare. [file 40900_2020_177_MOESM2_ESM.docx]

1. Which of the following best describe you (please check all that apply)

I am a retinoblastoma survivor

When were you diagnosed with retinoblastoma?

10 – 19 years ago

20 – 29 years ago

30 – 39 years ago

40 – 49 years ago

50 – 59 years ago

60 – 69 years ago

70 – 79 years ago

80 – 89 years ago

90 – 99 years ago

I am the mother or father of a child(ren) with retinoblastoma

How many of your children have been diagnosed with retinoblastoma?

1

2

3+

When was (were) your child(ren) first diagnosed with retinoblastoma? (If you have more than one child with retinoblastoma, please check all that apply.)

Less than 1 year ago

1 – 5 years ago

6 – 10 years ago

10+ years ago

I am a retinoblastoma researcher

I am a retinoblastoma healthcare provider

Other: __________________________________

2. How did you hear about this meeting?

3. Please circle the number below that best represents how you feel about each statement.

| **STATEMENT** | **STRONGLY AGREE** | **SOMEWHAT AGREE** | **NEUTRAL** | **SOMEWHAT DISAGREE** | **STRONGLY DISAGREE** |
| --- | --- | --- | --- | --- | --- |
| Retinoblastoma research is only relevant to clinicians. | 1 | 2 | 3 | 4 | 5 |
| Retinoblastoma clinicians lack the knowledge or skills needed to use research in their practice. | 1 | 2 | 3 | 4 | 5 |
| Retinoblastoma research is not relevant to the day-to-day lives of patients. | 1 | 2 | 3 | 4 | 5 |
| All patients should be given the opportunity to learn about, and participate in, retinoblastoma research. | 1 | 2 | 3 | 4 | 5 |
| Patients are encouraged to be involved in retinoblastoma research. | 1 | 2 | 3 | 4 | 5 |
| I can have a meaningful impact on retinoblastoma research. | 1 | 2 | 3 | 4 | 5 |

4. Patients & families can be involved in retinoblastoma research in the following ways (please check all that apply):

As study participants

As part of a research team

By setting research priorities and questions

By funding research

Other (please specify): ____________________

1. Please circle the number below that best represents how you feel about each statement.

| **STATEMENT** | **STRONGLY AGREE** | **SOMEWHAT AGREE** | **NEUTRAL** | **SOMEWHAT DISAGREE** | **STRONGLY DISAGREE** |
| --- | --- | --- | --- | --- | --- |
| Retinoblastoma research is only relevant to clinicians. | 1 | 2 | 3 | 4 | 5 |
| Retinoblastoma clinicians lack the knowledge or skills needed to use research in their practice. | 1 | 2 | 3 | 4 | 5 |
| Retinoblastoma research is not relevant to the day-to-day lives of patients. | 1 | 2 | 3 | 4 | 5 |
| All patients should be given the opportunity to learn about, and participate in, retinoblastoma research. | 1 | 2 | 3 | 4 | 5 |
| Patients are encouraged to be involved in retinoblastoma research. | 1 | 2 | 3 | 4 | 5 |
| I can have a meaningful impact on retinoblastoma research. | 1 | 2 | 3 | 4 | 5 |

2. Patients & families can be involved in retinoblastoma research in the following ways (please check all that apply):

As study participants

As part of a research team

By setting research priorities and questions

By funding research

Other (please specify): ____________________

| **Chart Board Questions** |
| --- |
| 1. What does patient-oriented research mean to you? |
| 2. What does patient engagement mean to you? |
| 3. How might we better engage patients and families in retinoblastoma research? |
| 4. Which of the following are your preferred sources of information about retinoblastoma research? Which are your actual sources of information about retinoblastoma research   \| INFORMATION SOURCE \| PLACE A STICKER ON YOUR PREFERRED SOURCES \| PLACE A STICKER ON YOUR ACTUAL SOURCES \| \| --- \| --- \| --- \| \| Physician \|  \|  \| \| Researcher \|  \|  \| \| Nurse \|  \|  \| \| Pharmacist \|  \|  \| \| Traditional Media: TV, Newspapers, Radio \|  \|  \| \| Online/Social Media \|  \|  \| \| Family & Friends \|  \|  \| \| Scientific Articles \|  \|  \| \| Pamphlets & Brochures \|  \|  \| \| I don’t get any information on retinoblastoma research \|  \|  \| \| Other ____________________ \|  \|  \| |

Each question was posted on large chart board paper around the meeting room. Members contributed responses using markers and Post-It notes.
