# Supplementary material for: The Canadian retinoblastoma research advisory board: a framework for patient engagement
Source: Res Involv Engagem. 2020 Feb 28;6:7. doi: 10.1186/s40900-020-0177-8 (PMC7048037; doi:10.1186/s40900-020-0177-8)
Supplement: Supplementary file 3 — Additional file 3: Second Annual CRRAB Meeting Evaluation. [file 40900_2020_177_MOESM3_ESM.docx]

**Canadian Retinoblastoma Research Advisory Board (CRRAB) – An Evaluation**

The upcoming Retinoblastoma Research Symposium is being hosted by the Canadian Retinoblastoma Research Advisory Board (CRRAB). CRRAB was founded in 2016, we invite you to provide feedback on the first year of CRRAB with this evaluation. The evaluation consists of 13 brief questions which should take no more than 15 minutes to complete.

1. Which best describes you? (check all that apply)
   1. Patient (Eg. Patient, family or friend of someone affected by retinoblastoma)
   2. Health professional (Eg. Physician, nurse, social worker, counselor)
   3. Researcher (Eg. Scientist, statistician, research coordinator)
2. Did you attend the first CRRAB meeting on December 3, 2016
   1. Yes
   2. No
3. Have you registered to attend the second CRRAB meeting on December 10, 2017
   1. Yes
   2. No
4. Which of the following CRRAB groups and activities are you aware of? (check all that apply)
   1. CRRAB Research Advisory Working Group
   2. CRRAB Patient Engagement Working Group
   3. CRRAB Priority Setting Working Group
   4. Canadian Retinoblastoma Research Registry
   5. Retinoblastoma Champion Program
   6. Retinoblastoma Priority Setting Study
   7. RB Patient Engagement Strategy Website ([www.rbresearch.ca](http://www.rbresearch.ca))
   8. RB Canada Research Blog ([www.rbcanadaresearch.com](http://www.rbcanadaresearch.com))
   9. RB Canada Research Email Blast
   10. CRRAB Twitter Account
   11. CRRAB Facebook Page
   12. None of the above
5. How did you first learn about CRRAB?
   1. A patient
   2. A health professional
   3. A researcher
   4. A pamphlet/letter
   5. Social Media
6. In which of the following CRRAB groups and activities did you participate in during 2017? (Check all that apply)
   1. CRRAB Research Advisory Working Group
   2. CRRAB Patient Engagement Working Group
   3. CRRAB Priority Setting Working Group
   4. Canadian Retinoblastoma Research Registry
   5. Retinoblastoma Champion Program
   6. Retinoblastoma Priority Setting Study
   7. None of the above
7. For how many months have you been involved in CRRAB activities?
   1. I have not been involved
   2. Less than 1 month
   3. 1-3 months
   4. 3-6 months
   5. 6-9 months
   6. 9-12 months
8. On a scale of 1-5 (1-never, 5-often), how often do you read/engage with the following CRRAB online media in 2017?

|  | 1 – Never | 2 – Rarely | 3 – Neutral | 4 - Sometimes | 5 - Often |
| --- | --- | --- | --- | --- | --- |
| RB Patient Engagement Strategy Website ([www.rbresearch.ca](http://www.rbresearch.ca)) |  |  |  |  |  |
| RB Canada Research Blog ([www.rbcanadaresearch.com](http://www.rbcanadaresearch.com)) |  |  |  |  |  |
| RB Canada Research Email Blast |  |  |  |  |  |
| CRRAB Twitter Account |  |  |  |  |  |
| CRRAB Facebook Page |  |  |  |  |  |

1. Beyond CRRAB, have you participated in any other patient engagement activities in 2017?

|  | Yes | No |
| --- | --- | --- |
| I participated in a research study as a subject |  |  |
| I was a team member on a research study |  |  |
| I was a team member on a grant application |  |  |
| I contributed to writing a scientific article |  |  |
| I contributed to writing a summary of scientific research |  |  |
| I attended an information session/workshop about patient engagement in research |  |  |
| I attended a conference with strong patient participation and inclusion |  |  |

Please use this space to elaborate on your patient engagement activities not covered by previous questions.

______________________________________________________________________________

1. On a scale of 1-5 (1 - strongly disagree, 5 – strongly agree), how would you rate the following statements?

|  | 1 –Strongly Disagree | 2 – Disagree | 3 – Neutral | 4 - Agree | 5 – Strongly Disagree |
| --- | --- | --- | --- | --- | --- |
| CRRAB engages a diverse group of retinoblastoma clinicians |  |  |  |  |  |
| CRRAB engages a diverse group of retinoblastoma researchers |  |  |  |  |  |
| CRRAB contributes to making research accessible to retinoblastoma survivors and family members |  |  |  |  |  |
| CRRAB encourages retinoblastoma survivors and family members to be involved in retinoblastoma research |  |  |  |  |  |
| Patient engagement in research will have a meaningful impact on retinoblastoma |  |  |  |  |  |

1. The objective of CRRAB is to create meaningful co-directed research that is co-directed with people affected by retinoblastoma. To this end, in 2017 we created a research registry and determined the top 10 retinoblastoma research priorities. What would you like CRRAB to accomplish in 2018?

______________________________________________________________________________

1. Please provide additional comments here.

____________________________________________________________________________

**Chart Board Discussions**

| **Chart Board Questions** |
| --- |
| 1. In 2018 CRRAB Should… |
| 1. Question 2: To have more patient and family leadership in CRRAB we should… |
| 1. I want to be part of CRRAB working groups because… |
| 1. I don’t want to be part of CRRAB working groups because… |
| 1. The purpose of CRRAB is to… |

Each question was posted on large chart board paper around the meeting room. Members contributed responses using markers and Post-It notes

**Post-Meeting Questionnaire**

**Canadian Retinoblastoma Research Advisory Board Annual Meeting**

**Meeting Evaluation Form**

| What were your objectives for the meeting? | Continue my involvement in CRRAB |  |
| --- | --- | --- |
|  | Meet individuals affected by retinoblastoma |  |
|  | Meet professionals interested in retinoblastoma |  |
|  | Share my story |  |
|  | Learn more about CRRAB |  |
|  | Other |  |
|  | If other, please specify: |  |

| Did you accomplish your objectives for the meeting? | Yes |  | No |  |
| --- | --- | --- | --- | --- |

What did you like most about the meeting?

What can we do better, next time?

|  |  |  |  |  |
| --- | --- | --- | --- | --- |
| Are the next steps for CRRAB clear? | Yes |  | No |  |
|  |  |  |  |  |
| Did you sign up to participate in a CRRAB Working Group? | Yes |  | No |  |

How likely are you to recommend CRRAB to others?

| Extremely Likely | Somewhat Likely | Neutral | Unlikely | Extremely Unlikely |
| --- | --- | --- | --- | --- |
|  |  |  |  |  |

Overall how satisfied were you with?:

|  | Very Satisfied | Satisfied | Neutral | Dissatisfied | Very Dissatisfied |
| --- | --- | --- | --- | --- | --- |
| Registration Process |  |  |  |  |  |
| Meeting Contents |  |  |  |  |  |
| Venue |  |  |  |  |  |
| Food and Beverage |  |  |  |  |  |
